# Supplementary material for: Characterisation and Distribution of Karaka Ōkahu Purepure Virus—A Novel Emaravirus Likely to Be Endemic to New Zealand
Source: Viruses. 2021 Aug 14;13(8):1611. doi: 10.3390/v13081611 (PMC8402849; doi:10.3390/v13081611)
Supplement: Supplementary file 1 [file viruses-13-01611-s001.zip › viruses-1295828-supplementary.pdf]

Supplementary Data

Supplementary Table S1. Percent identity between the amino acid sequences of P1 (the RdRp or L polymerase), P2 (the glycoprotein) and P3 (the nucleocapsid) of Karaka Ōkahu purepure virus (KŌPV) and known emaravirus orthologous sequences. Names and GenBank accession numbers for each viral sequence used in this study are given. The group to which each emaravirus has been designated in this study are also given.

Viral name	Emaravirus group	P1 Protein Accession Number	% identity P1 (RdRp)	P2 Protein Accession Number	% identity P2 (glycoprotein)	P3 Protein Accession Number	% identity P3 (Nucleoprotein)
Actinidia emaravirus 2	Ia	QEE82886	29.5	QEE82887	17.9	QEE82888	20.4
Aspen mosaic-associated virus	Ia	CAA0079389	29.3	CAA0079597	18.6	CAA0079646	18.9
Blackberry leaf mottle associated virus	Ia	AQX45473	28.7	AQX45474	19.3	QBM15204	23.9
Fig mosaic emaravirus	Ia	QBH72675	29.7	QTZ21228	19.6	QRN67942.1	23.9
Maple mottle-associated virus	Ia	QOI17315	29.7	QOI17316	18.3	QOI17317.1	16
Pigeonpea sterility mosaic emaravirus 1	Ia	ANQ90714	29.4	ANQ90715	17.8	QBA83604.1	16.7
Pigeonpea sterility mosaic emaravirus 2	Ia	QBA83607	29.7	QBA83608	17.9	CCV01188.2	19.7
Pistacia emaravirus	Ia	QAR18002	28.8	QAR18003	19.0	QAR18004.1	16.9
Rose rosette emaravirus	Ia	QHZ99235	29.2	QID76079	19.4	QIB97957.1	16
Vitis emaravirus T1	Ia	BCS90320	29.9	BCS90321	17.4	BCS90322.1	17.1
Actinidia chlorotic ringspot-associated virus	Ib	YP 009507925	29.1	ALX00128	19.4	QBL56976	14.5
European mountain ash ringspot-associated emaravirus	Ib	VFU05375	30.2	CAD7070703	21.5	CDL74499.1	19.3
Lilac chlorotic ringspot-associated virus	Ib	QIN85945	29.8	QIN85946	16.7	QIN85947	15.9
Redbud yellow ringspot-associated emaravirus	Ib	YP 009508083	29.0	AEO95761	17.9	YP_009508085	16.2
Common oak ringspot-associated virus	Ic	CAD0281685	28.8	CAD0281686	21.5	CAD0281686	26

High Plains wheat mosaic emaravirus	Ic	YP 009237277	26.9	QGT41788	12.4	QGT41068.1	18.1
Jujube yellow mottle-associated virus	Ic	QDM38999	27.3	QRG35046	17.4	QDM39001.1	18.5
Palo verde broom virus	Ic	AWH90165	28.0	AWH90172	18.9	AWH90177	18.2
Raspberry leaf blotch emaravirus	Ic	YP 009237274	27.4	CBZ42025	19.1	QCY49488	17.8
Ti ringspot-associated emaravirus	Ic	QAB47307	28.1	QAB47308	17.8	QAB47309.1	24.3
Chrysanthemum mosaic-associated virus	II	BCK60941	46.0	BCK60942	31.9	BCK60944	37.6
Pear chlorotic leaf spot-associated	II	BCG62015	44.4	QKY7700	30.3	QKY77005	26.2
Camellia japonica associated emaravirus 1	III	QGX73503	25.3	QGX73504	22.5	QGX73505.1	23.6
Camellia japonica associated emaravirus 2	III	QGX73507	26.2	QGX73508	21.0	QGX73509.1	20.4
Perilla mosaic virus	III	BBM96177	24.9	BBM96178	18.1	BBM96180	20

Supplementary Table S2. Primers used in this research.

Primer Name	Nucleotide sequence 5’ → 3’	Expected product size (bp)
3' RACE Gene Specific Primers		
3’RACE RNA 1A	TAC CAT GTA TGA GAT TCT GAA CC	~315
3’RACE RNA 1B	CTC ATC TTC AAA GAT ATC AAT GGG	~341
3’RACE RNA 2A	CAC CCA TTA TGC TGT TAG ATG	~300
3’RACE RNA 2B	AGT TGA CTC AGA GGT ATT CC	~370
3’RACE RNA 3A-2	CAT ACT GGT GAG TTC CTC C	~380
3’RACE RNA 3B-2	CAT CAA TTC ACG AGT TTG GG	~300
3’RACE RNA 4A	CCA TAT AAG TCA CTA CAT CTC AC	~290
3’RACE RNA 4B	TCA TTG TAT TTT ATT ATG CTT GGT TG	~430
3’RACE RNA 5A	TCC TTC TTG AGC TTA GTC AT	~300
3’RACE RNA 5B	GAA GAA GAG ACA ATA GAG TAG AGA	~240
PolyA Tail Primers		
PV1/SP6	GAT TTA GGT GAC ACT ATA GTTTTTTTTTTTTTTTTTV	
SP6	CAT ACG ATT TAG GTG ACA CTA TAG	

Target Segment	Nucleotide sequence 5' → 3'	Expected product size (bp)	Uniplex (U) or Multiplex (M)	Reference
KOPV Primers				
KOPV RNA 1 4669F	AGA AAT CAA CAG CTC ATC CAG AG	500	U, M	Unpublished
KOPV RNA 1 5168R	ATC AAC AGA GCT CAG ATG ACC TG			
KOPV RNA 2 482F	ACT AAG AGT TTG TCA TAT TTG TAA TCT ACC	441	U, M	Unpublished
KOPV RNA 2 922R	AGT TGA ACA AGA GCA CAT GAC CAC			
KOPV RNA 3 390F	AAC TGG GTT CCT TTA CTG GTG AC	504	U, M	Unpublished
KOPV RNA 3 863R	CTG AGA GCA TGA AGT CAA CAG CA			
KOPV RNA 4 169F *	GCA GAT TGC CTT ATT GCT TAT CAC C	481	U, M	Unpublished
KOPV RNA 4 649R *	TCA AGA TTG TGA TAT GAG GAT GGC A			
KOPV RNA 5 110F *	ATG ATT CTG TTA GGT CTG GTT GCT	500	U, M	Unpublished
KOPV RNA 5 609R *	AAT GAA AGC CTA CCC ATA CTT CCC			
Vv-NAD5-F	GCT TCT TGG GGC TTC TTG TTC G	220	U	(Chooi et al., 2013)
Vv-NAD5-R	TCC CTC CCA TCC CAG GAA TAA TTG			

Supplementary Table S3A and S3B. Nucleotide and amino acid length comparison of emaraviruses to date.

A.

RNA	Virus	Virus ID - Accession	Genome	3’ UTR	ORF	ORF	5' UTR
			Length (nt)	Length (nt)	Length (nt)	Length (aa)	Length (nt)
RNA 1	KOPV	MZ391827	7141	63	6963	2320	115
(RdRp)	AcCRaV	KT861481	7061	38	6912	2303	111
	AsMaV	LR742461	7106	45	6912	2303	149
	BLMaV	KY056657	7050	42	6915	2304	93
	ChMaV	LC576445	7093	64	6909	2302	120
	CORaV	LR828198	7109	58	6954	2317	97
	CRSaV-1	MN385573	7109	68	6963	2320	78
	CRSaV-2	MN385577	7120	69	6975	2324	76
	EMARaV	NC_013105	7040	44	6882	2293	114
	FMV	NC_029562	7039	39	6894	2297	106
	HPWMoV	NC_029570	6981	68	6819	2272	94
	JYMaV	MK305894	7143	47	6984	2327	112
	LiCRaV	MT112174	7047	54	6897	2298	96
	MaMaV	MT879190	7074	48	6918	2305	108
	Palo verde	MF766024	7015	60	6849	2282	106
	PCLSaV	LC554756	7099	68	6945	2314	86
	PerMV	LC496090	7291	79	7008	2335	204
	PiVB	MH727572	7027	52	6900	2299	75
	PPSMV1	HF568801	7022	49	6885	2294	88
	PPSMV2	NC_030660	7009	45	6885	2294	79
	RLBV	NC_029567	7062	48	6888	2295	126
	RRV	NC_015298	7026	62	6876	2291	88
	RYRSaV	JF795479	7049	40	6900	2299	109
	TiRSaV	MH223635	7217	56	7023	2340	138

	Vitis emaravirus T1	LC604727	7053	54	6903	2300	96
RNA 2 (Glycoprotein)	KOPV	MZ391828	1943	58	1800	599	85
	AcCRaV	KT861482	2267	54	1962	653	251
	AsMaV	LR742462	2288	57	1926	641	305
	BLMaV	KY056658	2271	47	1959	652	265
	ChMaV	LC576446	2054	70	1902	633	82
	CORaV	LR828199	2310	71	1956	651	283
	CjaEV1	MN385574	2054	69	1956	651	29
	CjaEV2	MN385578	2089	78	1983	660	28
	EMARaV	NC_013106	2335	58	1941	646	336
	FMV	NC_029565	2252	52	1926	641	274
	HPWMoV	NC_029549	2211	79	2004	667	128
	JYMaV	MK305895	2233	21	2022	673	190
	LiCRaV	MT112175	2268	41	1953	650	274
	MaMaV	MT879191	2289	55	1941	646	293
	Palo verde	MF766029	2098	40	1929	642	129
	PCLSaV	LC554757	2045	71	1839	612	135
	PerMV	LC496091	2092	54	1920	639	118
	PiVB	MH727573	2245	56	1923	640	266
	PPSMV1	HF568802.3	2223	41	1947	648	235
	PPSMV2	NC_030662	2229	47	1950	649	232
	RLBV	NC_029558	2135	49	1953	650	133
	RRV	NC_015299	2245	50	1938	645	257
	RYRSaV	JF795480	2220	50	1929	642	241
	TiRSaV	MH223636	2399	57	2154	717	188
	Vitis emaravirus T1	LC604728	2091	56	1917	638	118
RNA 3 (Nucelocapsid)	KOPV	MZ391829	1479	41	924	307	514
	AcCRaV	KT861483	1678	100	933	310	645
	AsMaV	LR742463	1587	100	957	318	530
	BLMaV	KY056659	1510	97	951	316	462
	ChMaV	LC576447	1390	74	792	263	524

	CORaV	LR828200	1338	74	876	291	388
	CjaEV1	MN385575	1360	115	888	295	357
	CjaEV2	MN385579	1316	116	900	299	300
	EMARaV	NC_013108	1559	119	945	314	495
	FMV	NC_029563	1491	99	948	315	444
	HPWMoV	NC_029550	1441	219	870	289	352
	JYMaV	MK305896	1259	70	885	294	304
	LiCRaV	MT112176	1557	88	930	309	539
	MaMaV	MT879192	1525	103	903	300	519
	Palo verde	MF766034	1356	85	879	292	392
	PCLSaV	LC554758	1250	88	801	266	361
	PerMV	LC496092	1080	92	798	265	190
	PiVB	MH727547	1525	101	918	305	506
	PPSMV1	HF568803	1442	102	927	308	413
	PPSMV2	NC_030661	1335	295	939	312	101
	RLBV	NC_029559	1365	53	879	292	433
	RRV	NC_015300	1544	99	951	316	494
	RYRSaV	JF795481	1414	89	942	313	383
	TiRSaV	MH223637	1106	128	861	286	117
	Vitis emaravirus T1	LC604729	1211	108	939	312	164
RNA 4 (Movement)	KOPV	MZ391830	1518	102	954	317	462
	AcCRaV	KT861484	1664	98	1140	379	426
	AsMaV	LR742464	1557	86	1086	361	385
	BLMaV	KY056660	1504	29	1143	380	332
	ChMaV	LC576449	1303	70	924	307	309
	CORaV	LR828201	1411	67	1098	365	246
	CjaEV1	MN385576	1349	154	1023	340	172
	CjaEV2	MN385580	1154	89	1026	341	39
	EMARaV	LR536379	1629	109	1122	373	398
	FMV	NC_029564	1472	81	1086	361	305
	HPWMoV	NC_029551	1682	112	1095	364	475
	JYMaV	MK305897	1547	101	1125	374	321
	LiCRaV	MT112177	1657	95	1146	381	416

MaMaV	MT879193	1533	87	1092	363	354
Palo verde	MF766039	1481	70	1101	366	310
PCLSaV	LC554759	1555	113	966	321	476
PerMV	LC496094	1294	102	1032	343	160
PiVB	MH727575	1550	81	1086	361	383
PPSMV1	HF568804	1563	77	1086	361	400
PPSMV2	NC_029560	1833	306	1422	473	105
RLBV	NC_029560	1675	81	1122	373	472
RRV	NC_015301	1541	83	1086	361	372
RYRSaV	JF795482	1513	99	1131	376	283
TiRSaV	MH223638	1342	81	999	332	262
Vitis emaravirus T1	LC604730	1628	80	1092	363	456

B.

RNA	Virus	Virus ID - Accession	RNA #	Genome	5' UTR	ORF	ORF	3' UTR
				Length (nt)	Length (nt)	Length (nt)	Length (aa)	Length (nt)
RNA 5 - 8 Unknown Function	KOPV	MZ391831	RNA 5	1576	86	810	269	680
	AcCRaV	KT861485	RNA 5	1476	75	702	233	699
	AsMaV	LR742465	RNA 5	1324	69	744	247	511
	BLMaV	KY056661	RNA 5	1224	68	681	226	475
	ChMaV	LC576450	RNA 5	1154	56	525	174	573
		LC576451	RNA 6	1707	84	1461	486	162
	CORaV	LR828202	RNA 5	1030	87	540	179	403
	CjaEV1	MN557024	RNA 5	1246	175	552	183	519
		MN557025	RNA 6	1474	124	606	201	744

	MN557026	RNA 7	1297	41	654	217	602
	MN557027	RNA 8	1335	41	654	217	640
	>MN557028	RNA 9	1155	113	858	285	184
EMARaV	NC_013107	RNA 4	1348	145	699	232	504
	LR536380	RNA 6	1362	147	690	229	525
FMV	NC_029566	RNA 5	1752	57	1509	502	186
	NC_029568	RNA 6	1212	68	567	188	577
HPWMoV	NC_029552	RNA 5	1715	120	1437	478	158
	NC_029553	RNA 6	1752	118	1479	492	155
	NC_029554	RNA 7	1434	117	918	305	399
	NC_029555	RNA 8	1339	93	531	176	715
JYMaV	MK305898	RNA 5	1267	83	837	278	347
	MK305899	RNA 6	980	89	585	194	306
LiCRaV	MT112178	RNA 5	1269	92	684	227	493
MaMaV	MT879194	RNA 5	1825	92	1434	477	299
	MT879195	RNA 6	1179	70	696	231	413
Palo verde	-	-	-	-	-	-	-
PCLSaV	LC554760	RNA 5	1253	86	849	282	318
PerMV	LC496095	RNA 5	1066	72	831	276	163
	LC496096	RNA 6	1128	33	762	253	333
	LC496099	RNA 7	998	73	726	241	199
PiVB	MH727576	RNA 5	1716	87	1449	482	180
	MH727578	RNA 6	1340	68	723	240	549
	MH727579	RNA 7	1709	14	1515	504	180
PPSMV1	HF945448.2	RNA 5	1801	84	1422	473	295
	KX363891	RNA 6	1194	29	756	251	409
PPSMV2	NC_030658	RNA 5	1833	105	1422	473	306

	NC_030659	RNA 6	1194	29	756	251	409
RLBV	NC_029561	RNA 5	1718	67	1431	476	220
	NC_029571	RNA 6	1095	93	567	188	435
	NC_029572	RNA 7	1089	77	579	192	433
	NC_029573	RNA 8	1273	86	702	233	485
RRV	NC_034979	RNA 5	1665	61	1404	467	200
	NC_034980	RNA 6	1402	68	702	233	632
	NC_034981	RNA 7	1649	61	1398	465	190
RYRSaV	KU904300	RNA 5	1272	86	672	223	514
TiRSaV	MH223639	RNA 5	1323	69	516	171	738
Vitis emaravirus T1	LC604731	RNA 5	1324	91	768	255	465

Supplementary Table S4. Genome coverage of Illumina sequencing

Karaka Ōkahu purepure virus (KŌPV)

Karaka Ōkahu purepure virus RNA	Length (nt)	Reads mapped from MiSeq (300 nt)	Coverage MiSeq	Reads mapped from HiSeq (21 nt sRNA)	Coverage HiSeq
1	7141	1234	52	508311	1495
2	1943	387	60	96979	1048
3	1479	2516	510	809420	11493
4	1518	1118	221	740503	10244
5	1576	1292	246	301915	4023
